# Lactate dehydrogenase‐to‐albumin ratio: A superior inflammatory marker for predicting contrast‐associated acute kidney injury after percutaneous coronary intervention

**DOI:** 10.1002/clc.24219

**Published:** 2024-01-29

**Authors:** Ji‐Lang Zeng, Jun‐Han Chen, Li‐Wei Zhang, Li‐Chuan Chen, Wen‐Jia Liang, Zhebin You, Kai‐Yang Lin, Yansong Guo

**Affiliations:** ^1^ Department of Cardiology, Shengli Clinical Medical College of Fujian Medical University Fujian Provincial Hospital Fuzhou China; ^2^ Fujian Provincial Key Laboratory of Cardiovascular Disease, Fujian Provincial Center for Geriatrics Fujian Provincial Clinical Research Center for Severe Acute Cardiovascular Diseases Fuzhou China; ^3^ Fujian Heart Failure Center Alliance Fuzhou China; ^4^ Fujian Key Laboratory of Geriatrics, Department of Geriatric Medicine, Fujian Provincial Hospital, Fujian Provincial Center for Geriatrics Fujian Medical University Fuzhou Fujian China

**Keywords:** all‐cause mortality, contrast‐associated acute kidney injury, lactate dehydrogenase‐to‐albumin ratio, percutaneous coronary intervention

## Abstract

**Purpose:**

Inflammation is commonly considered a mechanism underlying contrast‐associated acute kidney injury (CA‐AKI). This study aimed to explore the predictive capability of the novel inflammatory marker lactate dehydrogenase‐to‐albumin ratio (LAR) for CA‐AKI following percutaneous coronary intervention (PCI), and further compare it with other common inflammatory biomarkers.

**Methods:**

This study enrolled 5,435 patients undergoing elective PCI. The primary outcome was CA‐AKI, and the secondary outcome was all‐cause mortality. All patients were grouped into three groups based on the LAR tertiles.

**Results:**

Three hundred fifteen patients (5.8%) experienced CA‐AKI during hospitalization. The fully adjusted logistic regression suggested a significant increase in the risk of CA‐AKI in LAR Tertile 3 (odds ratio [OR]: 2.51, 95% confidence interval [CI]: 1.68−3.83, *p* < .001) and Tertile 2 (OR: 2.11, 95% CI: 1.42−3.20, *p* < .001) compared to Tertile 1. Additionally, receiver operating characteristic (ROC) analysis demonstrated that LAR exhibited significantly superior predictive capability for CA‐AKI compared to other inflammatory biomarkers. Regarding the secondary outcome, multivariate COX regression analysis showed a positive correlation between elevated LAR levels and all‐cause mortality.

**Conclusion:**

In patients undergoing elective PCI, LAR was significantly independently associated with CA‐AKI, and it stood out as the optimal inflammatory biomarker for predicting CA‐AKI.

## Graphical abstract

Inflammation plays a significant role in the pathophysiology of contrast‐associated acute kidney injury (CA‐AKI). We revealed that the novel inflammatory marker lactate dehydrogenase to albumin ratio (LAR) was a valuable predictor for CA‐AKI, surpassing other common inflammatory markers.

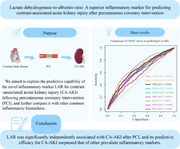

**Abbreviations**
AMI: acute myocardial infarction
CA‐AKI: contrast‐associated acute kidney injury
CHF: congestive heart failure
CKD: chronic kidney disease
dNLR: derived neutrophil‐to‐lymphocyte ratio
LAR: lactate dehydrogenase‐to‐albumin ratio
LDH: lactate dehydrogenase
MLR: monocyte‐to‐lymphocyte ratio
NGAL: neutrophil gelatinase‐associated lipocalin.
NLR: neutrophil‐to‐lymphocyte ratio
NPAR: neutrophil percentage‐to‐albumin ratio
PCI: percutaneous coronary intervention
PLR: platelet‐to‐lymphocyte ratio
PNI: prognostic nutritional index
RNS: reactive nitrogen species
ROS: reactive oxygen species
SCr: serum creatinine
SII: systemic immune‐inflammatory index
SIRI: systemic inflammation response index

## 1 INTRODUCTION

Over the past few decades, the widespread application of percutaneous coronary intervention (PCI) has significantly improved the survival and quality of life for patients with coronary artery disease. However, similar to many other medical interventions, PCI procedures are accompanied by a range of potential complications, with the most prevalent being contrast‐associated acute kidney injury (CA‐AKI). As the third leading cause of hospital‐acquired AKI, CA‐AKI bears a close relation to cardiovascular and renal adverse events.^1^, ^2^, ^3^ Compounding this, CA‐AKI presents a complex etiology, coupled with limited therapeutic choices, imposing substantial challenges on clinical practice.^4^, ^5^ Hence, there is a need to identify modifiable risk factors related to CA‐AKI, enabling the development of effective preventive strategies to reduce CA‐AKI incidence and improve outcomes.

Previous studies have emphasized the significant role of inflammatory response in the pathological processes of CA‐AKI. Furthermore, several inflammatory markers constructed based on serum parameters, such as systemic immune‐inflammation index (SII), neutrophil‐to‐lymphocyte ratio (NLR), and C‐reactive protein (CRP), have been extensively validated for their close associations with various inflammation‐associated disorders, including CA‐AKI.^6^, ^7^, ^8^, ^9^ Recently, a novel serum biomarker, known as the lactate dehydrogenase‐to‐albumin ratio (LAR), has been introduced to assess the inflammatory status in patients with various malignancies, and it has been confirmed to be closely linked to adverse prognosis.^10^, ^11^ Nevertheless, to date, there has been limited research into the potential association between LAR and CA‐AKI. Therefore, the present study aimed to investigate the predictive value of LAR for CA‐AKI as well as all‐cause mortality among patients treated with elective PCI.

## 2 MATERIALS AND METHODS

### 2.1 Study design and patients

In this retrospective, single‐center study, we consecutively enrolled 5841 patients after elective PCI at Fujian Provincial Hospital from January 2012 to December 2018. The exclusion criteria were listed as follows: (1) data for serum lactate dehydrogenase (LDH), albumin or serum creatinine (SCr) were missing (*n* = 325); (2) patients with severe liver dysfunction or renal insufficiency (estimated glomerular filtration rate [eGFR] less than 15 mL/min/1.73 m²) (*n* = 43); (3) patients who received contrast media or took nephrotoxic drugs within 1 week before or 48 h after the procedure (*n* = 21); (4) patients had a life expectancy of less than 1 year due to malignancy (*n* = 14); (5) patients died within 48 h after admission (*n* = 3). Ultimately, a total of 5435 patients were included in the final analysis. The study flow chart was illustrated in Figure S1.

### 2.2 Data collection

Upon admission, blood samples were routinely collected from all patients for biochemical analysis and other laboratory tests, such as LDH, albumin, and SCr. Meanwhile, SCr levels were continuously monitored for 2 days after contrast media exposure. The remaining patient data, including demographic information, medical history, comorbidities, angiographic parameters, and medication use were all obtained from the electronic medical records system.

### 2.3 PCI

The PCI procedure strictly adhered to the current AHA/ACC guidelines and was performed by a team of at least two experienced interventional cardiologists. During the procedure, all patients were administered nonionic, low‐osmolarity contrast media (either Ultravist or Iopamiron, both 370 mgI/mL). Moreover, patients received continuous intravenous infusions of 0.9% normal saline at a rate of 1 mL/kg/h for a duration of 12 h throughout the perioperative period. For individuals with heart failure, the rate was reduced to 0.5 mL/kg/h.

### 2.4 Calculation of other inflammatory biomarkers

In addition to LAR (LDH [U/L]/albumin [g/L]), we also assessed several common serum inflammatory biomarkers in clinical research, including SII, systemic inflammation response index (SIRI), NLR, derived NLR (dNLR), neutrophil percentage‐to‐albumin ratio, platelet‐to‐lymphocyte ratio, monocyte‐to‐lymphocyte ratio, and prognostic nutritional index (PNI). The calculation formulas for these biomarkers were provided in Table S1.

### 2.5 Clinical outcomes and follow‐up

The primary outcome was set as CA‐AKI, defined as an increase in SCr ≥.3 mg/dL or ≥50% from baseline SCr within 48 h following the PCI procedure.^12^ The secondary outcome was all‐cause mortality. The follow‐up data were obtained from medical records of outpatient clinics or through telephone interviews. Finally, 233 (4.3%) patients were lost to follow‐up at the beginning of the study.

### 2.6 Statistical analysis

All patients were grouped into three groups based on the tertiles of LAR levels: Tertile 1 (low LAR group, LAR < 3.76), Tertile 2 (middle LAR group, 3.76 ≤ LAR < 4.74), and Tertile 3 (high LAR group, LAR ≥ 4.74). Continuous variables following normal distribution were reported as mean ± standard deviation (SD), whereas those deviating from normality were denoted as median and interquartile range. Categorical variables were represented as count (percentage). Comparison of continuous variables was conducted using analysis of variance test (normal distribution) or Kruskal‐Wallis test (skewed distribution). For categorical variables, differences between groups were assessed using the *χ* ^2^ test or Fischer's exact test.

Restricted cubic spline (RCS) curves were performed to assess the potential nonlinear association between LAR and CA‐AKI. To identify potential risk factors for CA‐AKI, we conducted univariate logistic regression analysis. Variables with a significance level (*p* < .05) or those considered clinically significant were subsequently incorporated into the multivariate logistic regression models. Specifically, these multivariate models were defined as follows: In Model 1, we made adjustments for age >75 years and sex. Building upon Model 1, Model 2 further incorporated diabetes mellitus, hypertension, chronic kidney disease (CKD), congestive heart failure (CHF), and hypotension. Finally, Model 3 expanded upon Model 2 by including acute myocardial infarction (AMI), anemia, and contrast media > 150 mL. To evaluate multicollinearity among the covariates, we utilized the variance inflation factor (VIF) method. Multicollinearity was considered to be present if the VIF value was ≥5. Subsequently, we conducted subgroup analyses to ascertain whether there were discrepancies in the association between LAR and CA‐AKI among various subgroups, simultaneously calculating the *p* values for interactions. We also adopted Receiver Operating Characteristic (ROC) curves and DeLong tests to evaluate the predictive performance of LAR relative to other inflammatory markers in predicting CA‐AKI. Additionally, we calculated the Spearman correlation coefficients to calculate the correlation between LAR and other inflammatory markers. Subsequently, we conducted multivariate COX regression analysis to explore the relationship between LAR and all‐cause mortality. Survival differences among the three groups were compared using Kaplan−Meier curves and log‐rank tests. All statistical analyses were done using R software (version 4.3.0; R Foundation). A two‐tailed *p* < .05 suggested statistical significance.

## 3 RESULTS

### 3.1 Baseline characteristics of the study population

The study population had an average age of 65.4 ± 10.3 years, and 1173 individuals (21.6%) were female. Table 1 delineated the baseline characteristics of patients grouped according to the LAR tertiles. As illustrated in Table 1, patients with higher LAR levels tended to be older and had a higher proportion of females. In terms of comorbidities, there was a progressive rise in the occurrence of AMI, CKD, and anemia with increasing LAR tertiles (all *p* < 0.05). Moreover, compared with patients in Tertile 1, those in Tertile 3 had lower albumin, eGFR, and left ventricular ejection fraction. Conversely, they exhibited higher levels of white blood cell counts, cholesterol, and D‐dimer. Simultaneously, individuals with elevated LAR levels showed a higher likelihood of CHF and, as a result, received diuretics treatment more frequently. More importantly, patients in Tertile 3 displayed the highest degree of inflammation, as indicated by their elevated levels of inflammation markers (such as SII and SIRI). Further correlation analysis showed a significant relationship between LAR and other inflammatory markers (all *p* < 0.001) (Table S2). Table S3 outlined the baseline characteristics of patients with and without CA‐AKI.

**Table 1:** Baseline characteristics of the study population grouped according to the LAR tertiles.

	Tertile 1 (Low LAR)	Tertile 2 (Middle LAR)	Tertile 3 (High LAR)	*p* Value
Number of patients	*n* = 1812	*n* = 1811	*n* = 1812	
*Demographic information*				
Age, mean ± SD, years	62.9 ± 10.2	66.5 ± 9.4	66.7 ± 10.9	<.001
Age >75 years, *n* (%)	210 (11.6)	316 (17.5)	410 (22.6)	<.001
Sex, female, *n* (%)	313 (17.3)	434 (24.0)	426 (23.5)	<.001
BMI, median (IQR), kg/m^2^	24.2 (22.4−26.1)	24.4 (22.5−26.4)	24.2 (22.1−26.4)	.104
*Comorbidities, n (%)*				
Diabetes mellitus	681 (37.6)	638 (35.2)	623 (34.4)	.114
Hypertension	1186 (65.5)	1288 (71.1)	1219 (67.3)	.001
Congestive heart failure	33 (1.8)	57 (3.2)	151 (8.3)	<.001
Chronic kidney disease	106 (5.9)	152 (8.4)	229 (12.6)	<.001
Anemia	73 (4.0)	136 (7.5)	295 (16.3)	<.001
AMI	194 (10.7)	295 (16.3)	1168 (64.5)	.001
Hypotension	75 (4.1)	86 (4.8)	205 (11.3)	<.001
*Laboratory tests, median (IQR)*				
White blood cell, 10^9^/L	6.79 (5.70−7.92)	6.77 (5.70−7.97)	7.54 (6.02−9.53)	<.001
Hemoglobin, g/L	142 (132−151)	138 (128−148)	134 (122−145)	<.001
Platelet, 10^9^/L	213 (183−251)	212 (179−253)	213 (178−256)	.667
Neutrophil, 10^9^/L	4.08 (3.31−5.00)	4.08 (3.24−5.05)	4.96 (3.69−6.71)	<.001
Neutrophil percentage, %	62.9 (56.8−69.2)	62.9 (56.6−68.8)	67.4 (61.0−73.4)	<.001
Monocyte, 10^9^/L	0.44 (0.35−0.55)	0.44 (0.34−0.55)	0.53 (0.40−0.70)	<.001
Lymphocyte, 10^9^/L	1.93 (1.55−2.41)	1.88 (1.53−2.33)	1.69 (1.32−2.13)	<.001
Creatinine, mg/dL	0.87 (0.76−1.00)	0.88 (0.76−1.02)	0.88 (0.75−1.04)	.209
eGFR, mL/min/1.73m^2^	94 (82−101)	91 (76−99)	90 (73−99)	<.001
LDH, U/L	144 (133−155)	174 (163−187)	253 (212−405)	<.001
Albumin, g/L	43 (41−46)	42 (40−44)	39 (36−42)	<.001
Cholesterol, mmol/L	3.97 (3.33−4.80)	4.03 (3.41−4.88)	4.19 (3.54−5.04)	<.001
LDL‐C, mmol/L	2.45 (1.88−3.14)	2.54 (1.97−3.28)	2.71 (2.15−3.39)	<.001
Glucose, mmol/L	5.48 (4.98−6.50)	5.43 (4.91−6.51)	5.49 (4.91−6.78)	.224
HbA1c, %	6.2 (5.8−6.9)	6.2 (5.8−7.1)	6.2 (5.8−7.0)	.330
d‐dimer, mg/L FEU	0.26 (0.16−0.45)	0.32 (0.20−0.60)	0.45 (0.24−0.89)	<.001
NT‐proBNP, pg/mL	84 (39−196)	164 (68−469)	785 (252−2121)	<.001
LVEF < 40, *n* (%)	24 (1.4)	37 (2.2)	79 (4.6)	<.001
LVEF, %	60 (58−63)	60 (57−62)	58 (54−61)	<.001
LAR	3.36 (3.11−3.56)	4.16 (3.95−4.40)	6.60 (5.29−10.52)	.001
SII	442 (326−616)	450 (323−642)	630 (409−954)	<.001
SIRI	0.91 (0.63−1.30)	0.93 (0.63−1.41)	1.51 (0.94−2.71)	<0.001
NLR	2.06 (1.58−2.76)	2.11 (1.62−2.86)	2.92 (2.06−4.26)	<.001
dNLR	1.54 (1.21−2.02)	1.57 (1.23−2.03)	2.01 (1.52−2.80)	<.001
NPAR	14.4 (12.9−16.2)	15.0 (13.3−16.8)	17.3 (15.2−19.5)	<.001
PLR	110 (88−139)	114 (90−142)	125 (97−167)	<.001
MLR	0.22 (0.17−0.29)	0.23 (0.18−0.31)	0.31 (0.23−0.45)	<.001
PNI	53 (50−57)	52 (48−55)	48 (44−51)	<.001
*Medication use during hospitalization, n (%)*				
Antiplatelet agents	1810 (99.9)	1806 (99.7)	1810 (99.9)	.453
Statin	1796 (99.1)	1799 (99.3)	1795 (99.1)	.626
ACEI/ARB	1478 (81.6)	1503 (83.0)	1514 (83.5)	.265
β‐blocker	1511 (83.4)	1506 (83.2)	1478 (81.6)	.288
Diuretics	218 (12.0)	339 (18.7)	780 (43.1)	<.001
*Angiographic parameters, mean ± SD*				
Multivessel disease, *n* (%)	1388 (80.2)	1473 (84.6)	1499 (85.2)	<.001
Stent length, median (IQR), mm	38 (23−60)	38 (23−61)	38 (23−63)	.054
Number of stents, median (IQR), *n*	1 (1−2)	2 (1−2)	2 (1−2)	.270
Contrast media, mL	192.94 ± 64.02	195.96 ± 64.16	198.21 ± 64.53	.063

Abbreviations: ACEI/ARB, angiotensin‐converting enzyme inhibitor/angiotensin receptor blocker; AMI, acute myocardial infarction; BMI, body mass index; dNLR, derived neutrophil‐to‐lymphocyte ratio; eGFR, estimated glomerular filtration rate; HbA1c (%), glycosylated hemoglobin; LAR, lactate dehydrogenase‐to‐albumin ratio; LDH, lactate dehydrogenase; LDL‐C, low density lipoprotein‐cholesterol; LVEF, left ventricular ejection fraction; MLR, monocyte‐to‐lymphocyte ratio; NLR, neutrophil‐to‐lymphocyte ratio; NPAR, neutrophil percentage‐to‐albumin ratio; NT‐proBNP, N‐terminal pro‐brain natriuretic peptide; PLR, platelet‐to‐lymphocyte ratio; PNI, prognostic nutritional index; SII, systemic immune‐inflammatory index; SIRI, systemic inflammation response index.

### 3.2 Association between LAR and CA‐AKI

**Figure 1:**
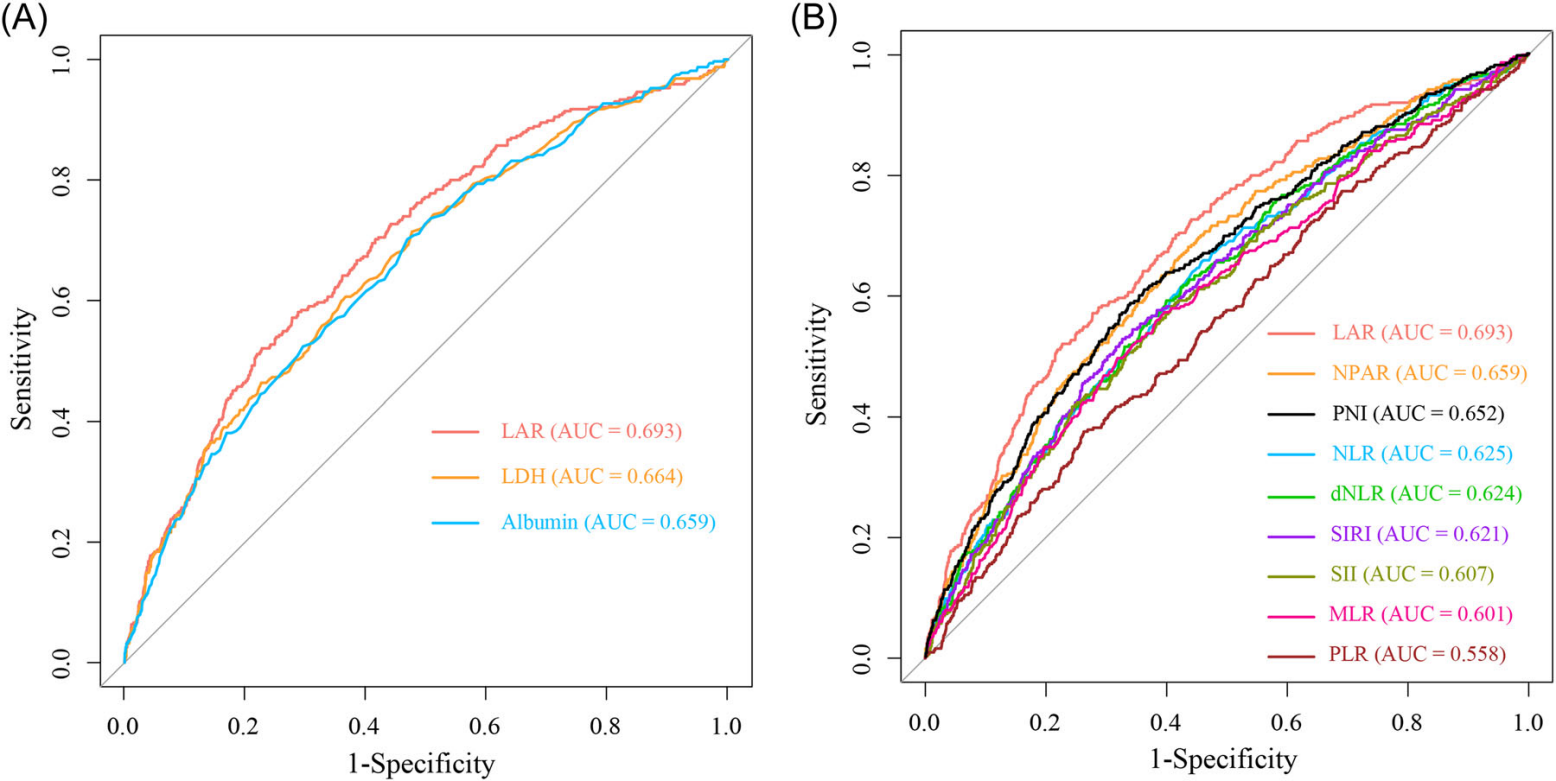
(A) Comparison of the predictive abilities of LAR, LDH, and albumin for CA‐AKI; (B) Comparison of predictive abilities for CA‐AKI among LAR and other inflammatory markers. CA‐AKI, contrast‐associated acute kidney injury; LAR, lactate dehydrogenase‐to‐albumin ratio; LDH, lactate dehydrogenase; Other abbreviations were shown in Table 1.

**Figure 2:**
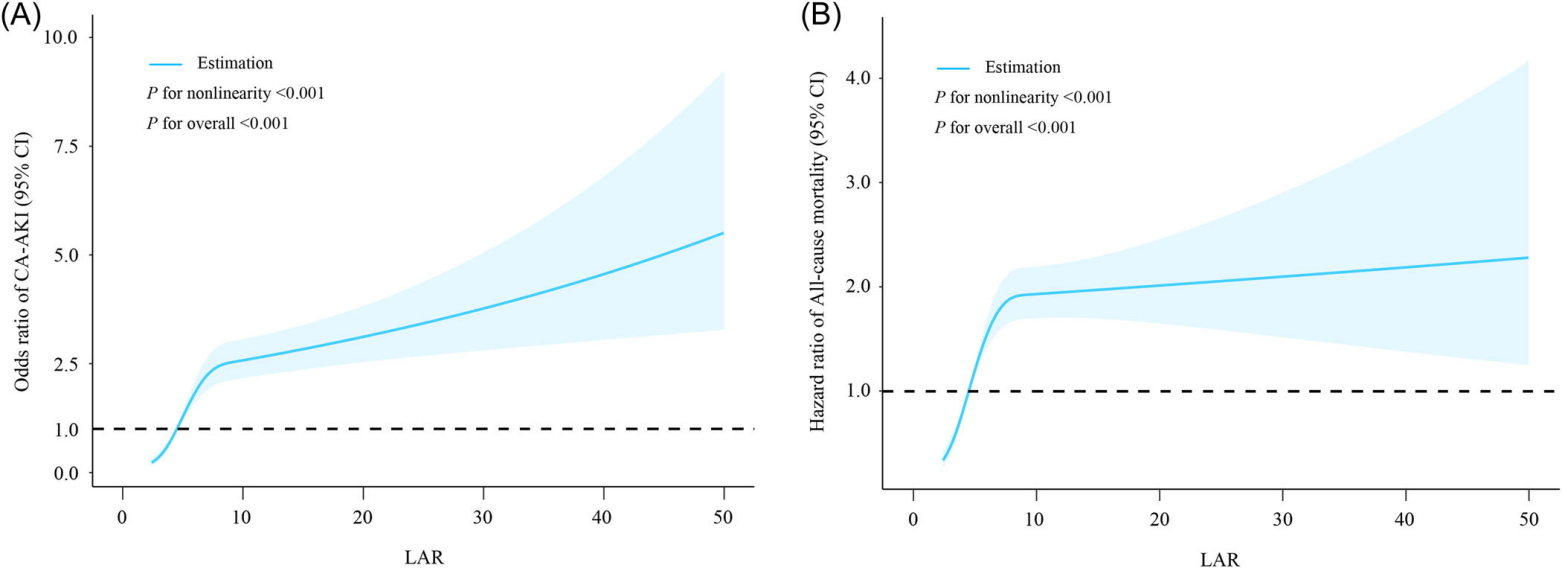
(A) dose–response relationship between LAR and the risk of CA‐AKI; (B) dose–response relationship between LAR and the risk of all‐cause mortality. The blue shaded area represents the 95% confidence interval. CA‐AKI, contrast‐associated acute kidney injury; LAR, lactate dehydrogenase‐to‐albumin ratio; Other abbreviations were shown in Table 1.

A total of 315 patients (5.8%) experienced CA‐AKI during hospitalization. The rates of CA‐AKI displayed an upward trend corresponding to increasing LAR tertiles, with rates of 2.2%, 4.9%, and 10.3% in Tertiles 1, 2, and 3, respectively (*p* for trend = .001) (Figure S2). The ROC curves illustrated that the predictive ability of LAR (Area under the curve [AUC] = 0.693, 95% confidence interval [CI]: 0.662−0.723) for CA‐AKI was significantly better than that of LDH (AUC = 0.664, 95% CI: 0.632−0.696; ΔAUC: 0.029, DeLong test *p* = .040) or albumin (AUC = 0.659, 95% CI: 0.628−0.691; ΔAUC = 0.034, DeLong test *p* = .031) (Figure 1A). The RCS curve demonstrated a nonlinear positive correlation between LAR and CA‐AKI (*p* for overall < 0.001, *p* for nonlinearity < .001) (Figure 2A).

As presented in Table 2, the fully adjusted logistic regression model (Model 3) further suggested that there was a notable increase in the risk of CA‐AKI in LAR Tertile 3 (odds ratio [OR]: 2.51, 95% CI: 1.68−3.83, *p* < .001] and Tertile 2 (OR: 2.11, 95% CI: 1.42−3.20, *p* < .001) compared to Tertile 1 (*p* for trend <.001). When analyzing LAR as a continuous variable, we obtained similar findings, with each 1‐SD increase in LAR corresponding to a 17% increment in the risk of CA‐AKI (fully adjusted OR: 1.17, 95% CI: 1.08−1.28, *p* < .001). The multicollinearity analysis revealed that there was no collinearity among covariates (all VIF values < 5). The results of subgroup analyses indicated that higher LAR levels were consistently linked to an increased risk of CA‐AKI, both in the AMI and non‐AMI subgroups (Figure S3). Furthermore, the effect of LAR on CA‐AKI was remarkably more significant within the AMI subgroup (*p* for interaction = .031). No significant interactions were observed in other subgroups (all *p* for interaction > .05).

**Table 2:** The odds ratio for the association of LAR with CA‐AKI.

	Each 1‐SD increase	LAR tertiles			*p* for trend
		Tertile 1	Tertile 2	Tertile 3	
Unadjusted model	1.38 (1.27−1.51)***	1 (Ref.)	2.26 (1.56−3.34)***	5.10 (3.64−7.31)***	<.001
Model 1	1.38 (1.27−1.51)***	1 (Ref.)	2.15 (1.48−3.17)***	4.72 (3.36−6.79)***	<.001
Model 2	1.33 (1.21−1.46)***	1 (Ref.)	2.09 (1.44−3.09)***	4.14 (2.93−5.98)***	<.001
Model 3	1.17 (1.08−1.28)***	1 (Ref.)	2.11 (1.42−3.20)***	2.51 (1.68−3.83)***	<.001

*Note*: *p < .05; **p < .01; ***p < .001.

Model 1: adjusted age > 75 years and sex.

Model 2: adjusted for Model 1 + diabetes mellitus, hypertension, chronic kidney disease, congestive heart failure, and hypotension.

Model 3: adjusted for Model 2 + acute myocardial infarction, anemia, and contrast media > 150 mL.

Abbreviations: CA‐AKI, contrast‐associated acute kidney injury; LAR, lactate dehydrogenase‐to‐albumin ratio.

### 3.3 Comparison of predictive value of LAR with other inflammatory biomarkers

ROC curve analyses revealed that LAR achieved the highest AUC among these nine inflammatory markers and was significantly superior to the other eight inflammatory markers (all Delong test *p* < .05) (Figure 1B and Table S4).

### 3.4 Association between LAR and all‐cause mortality

The median follow‐up duration was 35.8 months, during which 460 (8.8%) patients died. According to the RCS model, a nonlinear, positive relationship was observed between LAR and all‐cause mortality (*p* for overall < .001, *p* for nonlinearity < .001) (Figure 2B). Multivariate COX regression analysis confirmed that whether LAR was regarded as a continuous variable or a categorical variable, it exhibited significant independent predictive value for all‐cause mortality (Table 3). In addition, Kaplan−Meier survival curves produced similar results, indicating significant differences in survival among the three groups of patients (log‐rank test, *p* < .001) (Figure S4).

**Table 3:** The hazard ratio for the association of LAR with all‐cause mortality.

	Each 1‐SD increase	LAR tertiles			*p* for trend
		Tertile 1	Tertile 2	Tertile 3	
Unadjusted model	1.14 (1.10−1.19)***	1 (Ref.)	1.98 (1.51−2.60)***	3.42 (2.66−4.41)***	<.001
Model 1	1.15 (1.10−1.20)***	1 (Ref.)	1.76 (1.34−2.31)***	2.88 (2.23−3.72)***	<.001
Model 2	1.12 (1.07−1.17)***	1 (Ref.)	1.72 (1.31−2.25)***	2.52 (1.94−3.27)***	<.001
Model 3	1.10 (1.04−1.16)***	1 (Ref.)	1.68 (1.28‐−2.21)***	2.50 (1.87−3.33)***	<.001

*Note*: *p < .05; **p < .01; ***p < .001.

Model 1: adjusted age > 75 years and sex.

Model 2: adjusted for Model 1 + diabetes mellitus, hypertension, chronic kidney disease, congestive heart failure, and hypotension.

Model 3: adjusted for Model 2 + acute myocardial infarction, anemia, and contrast‐associated acute kidney injury.

Abbreviations: LAR, dehydrogenase‐to‐albumin ratio.

### 3.5 Sensitivity analysis

To ensure the reliability of our primary findings, we conducted a sensitivity analysis. Specifically, we adopted the definition of CA‐AKI as recommended by the European Society of Urogenital Radiology (ESUR), which defined CA‐AKI as an increase in SCr of ≥0.5 mg/dL or ≥25% from baseline within 48 h after exposure to contrast media.^13^ The results demonstrated good consistency (Table S5).

## 4 DISCUSSION

Our study provided new insights into the relationship between LAR and renal impairment after PCI. Firstly, ROC curve analysis demonstrated that LAR had a good diagnostic ability for CA‐AKI. Moreover, We observed a significant correlation between elevated LAR levels and an increased risk of CA‐AKI, even after adjusting for traditional risk factors. Meanwhile, our findings indicated that LAR can serve as an independent predictor of all‐cause mortality.

Although the exact mechanism linking LAR to CA‐AKI remains unclear, we may explore it from two perspectives: LDH and albumin. Firstly, LDH levels are closely intertwined with inflammation and oxidative stress. Oxidative stress is regarded as an additional potential pathogenic mechanism of CA‐AKI.^14^ It is widely recognized that inflammation and oxidative stress are interconnected and interdependent pathophysiological processes. Specifically, inflammatory cells release reactive oxygen species (ROS), which exacerbate oxidative damage, and reciprocally, ROS enhances the inflammatory response.^15^ These interactional processes can result in cellular damage and membrane rupture, subsequently causing the release of LDH. Furthermore, recent studies have observed that the application of LDH inhibitors or the knockout of the LDH gene can effectively mitigate cellular inflammation and oxidative damage.^16^, ^17^ Second, high LDH levels are widely accepted indicators of MI. After an MI, myocardial cell membranes become damaged and rupture, resulting in the release of LDH into the bloodstream. Simultaneously, reduced cardiac output causes a decrease in renal blood flow, which is aggravated by the vasoconstrictive effects of contrast media. This, in turn, worsens renal function and raises the likelihood of CA‐AKI. Our subgroup analysis also found that the association between LAR and CA‐AKI was more prominent in the AMI subgroup. Third, as one of the key enzymes in lactate metabolism, high LDH levels may indicate disruptions in lactate metabolism. Under normal circumstances, a small amount of lactate is a byproduct of glycolysis, with a portion cleared by the kidney.^18^ However, in the presence of metabolic disturbances (such as hypoxia), increased expression of LDH leads to a substantial rise in lactate produced through anaerobic glycolysis.^19^ Simultaneously, diminished renal clearance of lactate due to hypoxia results in its accumulation. Indeed, elevated lactate levels are often considered an indicator of inadequate organ perfusion.^20^ Hence, increased LDH levels may reflect inadequate renal perfusion, reducing the ability to handle contrast media and consequently increasing susceptibility to CA‐AKI.

Similar to LDH, albumin plays a crucial role in the context of inflammation and oxidative stress. It has been reported that albumin acts as a negative acute‐phase reactant, with its serum levels typically decreasing in situations involving inflammation or malnutrition.^21^, ^22^ Moreover, albumin has been demonstrated to exhibit anti‐inflammatory properties by interfering with the NF‐κB inflammatory signaling pathway.^23^ In addition, there is a negative relationship between albumin levels and levels of neutrophil gelatinase‐associated lipocalin (NGAL), which is commonly associated with inflammation and kidney injury and is recognized as a biomarker for renal function and damage.^24^, ^25^ Meanwhile, research has established that albumin can form complexes with transition metal ions such as iron and copper, thereby preventing these metal ions from participating in the Fenton reaction, which is responsible for generating ROS.^26^ Consequently, this mechanism helps mitigate oxidative damage. The antioxidant properties of albumin are also associated with its free radical‐trapping abilities. In the physiological state, albumin primarily exists in the form of human mercaptalbumin, containing multiple thiol groups with reducing power on its molecules. These thiol groups can capture various ROS and reactive nitrogen species (RNS), thereby exerting antioxidative effects.^26^, ^27^ Moreover, several retrospective studies have established the correlation between albumin and its derived parameters (such as PNI) with CA‐AKI.^28^, ^29^ Another recent comprehensive meta‐analysis, encompassing a cohort of nearly 20 000 patients, has reaffirmed that hypoalbuminemia was significantly linked to an increased risk of CA‐AKI incidence.^30^ Numerous observational studies have also highlighted the predictive and prognostic value of albumin in cardiovascular diseases.^22^, ^31^

To our best understanding, this was the first study to evaluate the relationship between LAR and CA‐AKI as well as all‐cause mortality after PCI. Traditionally, LDH is used to reflect tissue damage and the extent of inflammation, whereas albumin is closely linked to antioxidative functions and renal functionality. LAR combines information from both aspects, providing a more comprehensive representation of inflammatory status and potential kidney injury risk. This was confirmed in our study, wherein LAR significantly enhanced the predictive capability for CA‐AKI compared to individual measures of LDH and albumin. Moreover, the predictive efficacy of LAR surpasses that of other commonly inflammatory biomarkers. In summary, the evidence we presented indicates that LAR, as a simple and cost‐effective routine laboratory test, has the potential to serve as a powerful tool for the early identification of high‐risk CA‐AKI patients, thereby offering valuable guidance for clinical decision‐making.

There are some limitations to this study that need to be mentioned. Firstly, this study was a single‐center retrospective study, and thus the findings need to be further confirmed by multicenter prospective studies. Additionally, like any observational study, we cannot rule out the possibility of residual confounding affecting our study findings. Third, routine measurements of CRP were not conducted in our study population upon admission, limiting further analysis in this regard. Fourthly, levels of LDH and albumin were measured only once upon admission, without dynamic monitoring during hospitalization. This inevitably introduced random errors. Lastly, variations in the timing of measurements may result in missing the peak SCr levels after PCI, resulting in an underestimation of CA‐AKI incidence.

## 5 CONCLUSION

In patients undergoing elective PCI, LAR was significantly independently associated with CA‐AKI, and it stood out as the optimal inflammatory biomarker for predicting CA‐AKI.

## CONFLICT OF INTEREST STATEMENT

The authors declare no conflict of interest.

## Supporting information

Supporting information.

Supporting information.

Supporting information.

Supporting information.

Supporting information.

## Data Availability

Due to confidentiality reasons, the data set generated and analyzed during the current study is not publicly available. However, upon approval from the Ethics Committee of Fujian Provincial Hospital, it can be obtained from the corresponding author.
