# Supplementary figures and images for: Lactate dehydrogenase‐to‐albumin ratio: A superior inflammatory marker for predicting contrast‐associated acute kidney injury after percutaneous coronary intervention
Source: Clin Cardiol. 2024 Jan 29;47(2):e24219. doi: 10.1002/clc.24219 (PMC10823551; doi:10.1002/clc.24219)

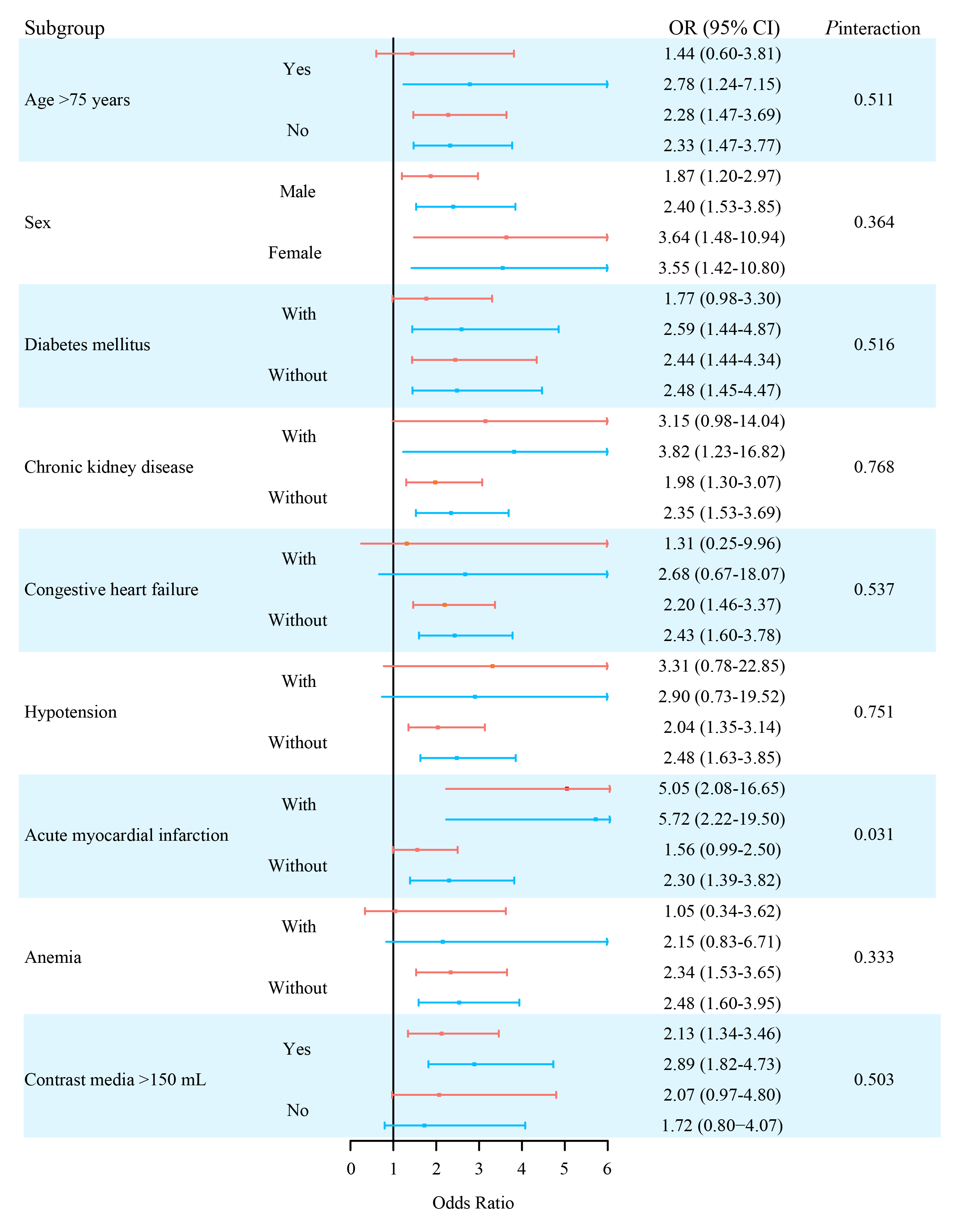

Supplement: Supplementary file 1 — Supporting information. [file CLC-47-e24219-s003.tif]

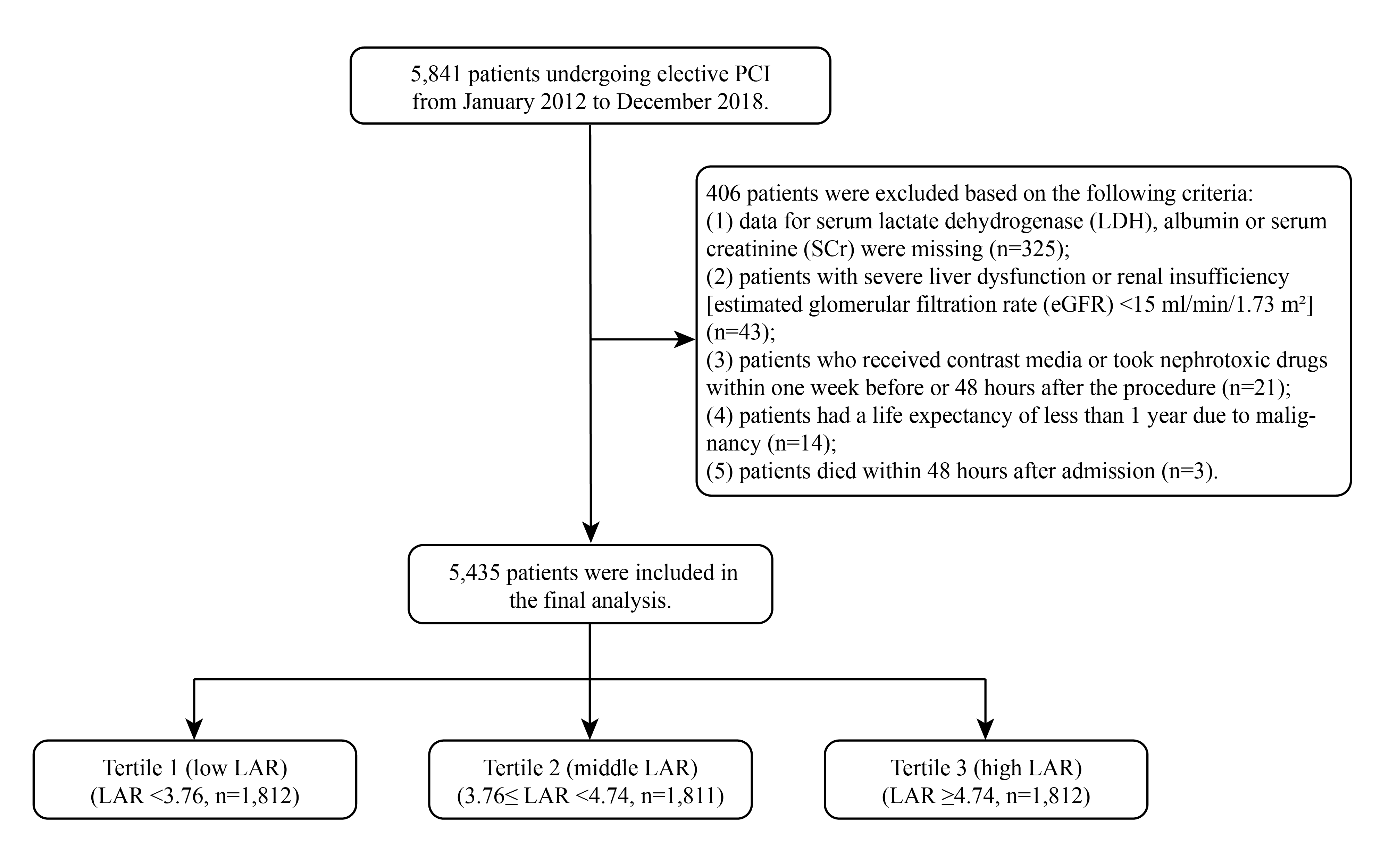

Supplement: Supplementary file 2 — Supporting information. [file CLC-47-e24219-s004.tif]

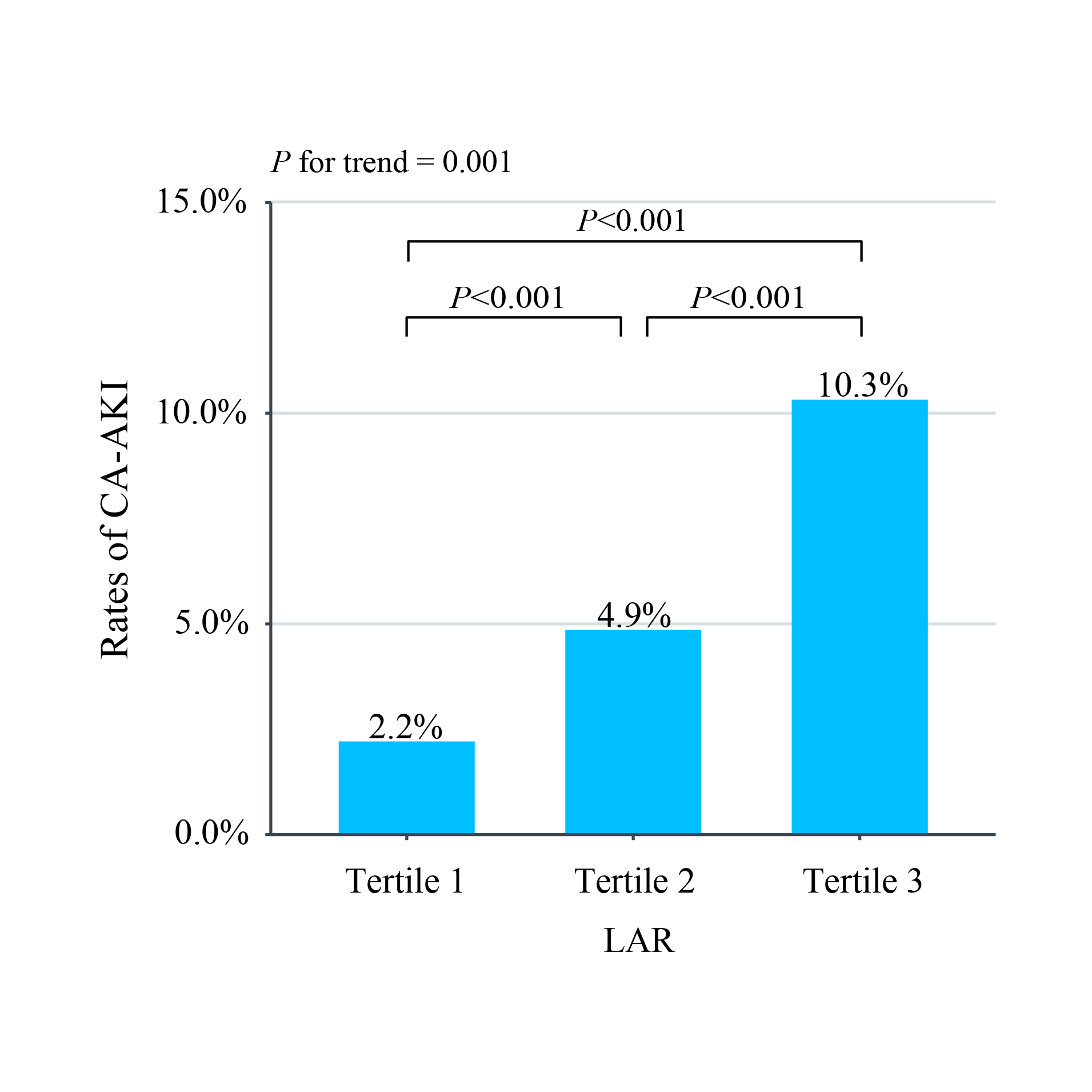

Supplement: Supplementary file 3 — Supporting information. [file CLC-47-e24219-s002.tif]

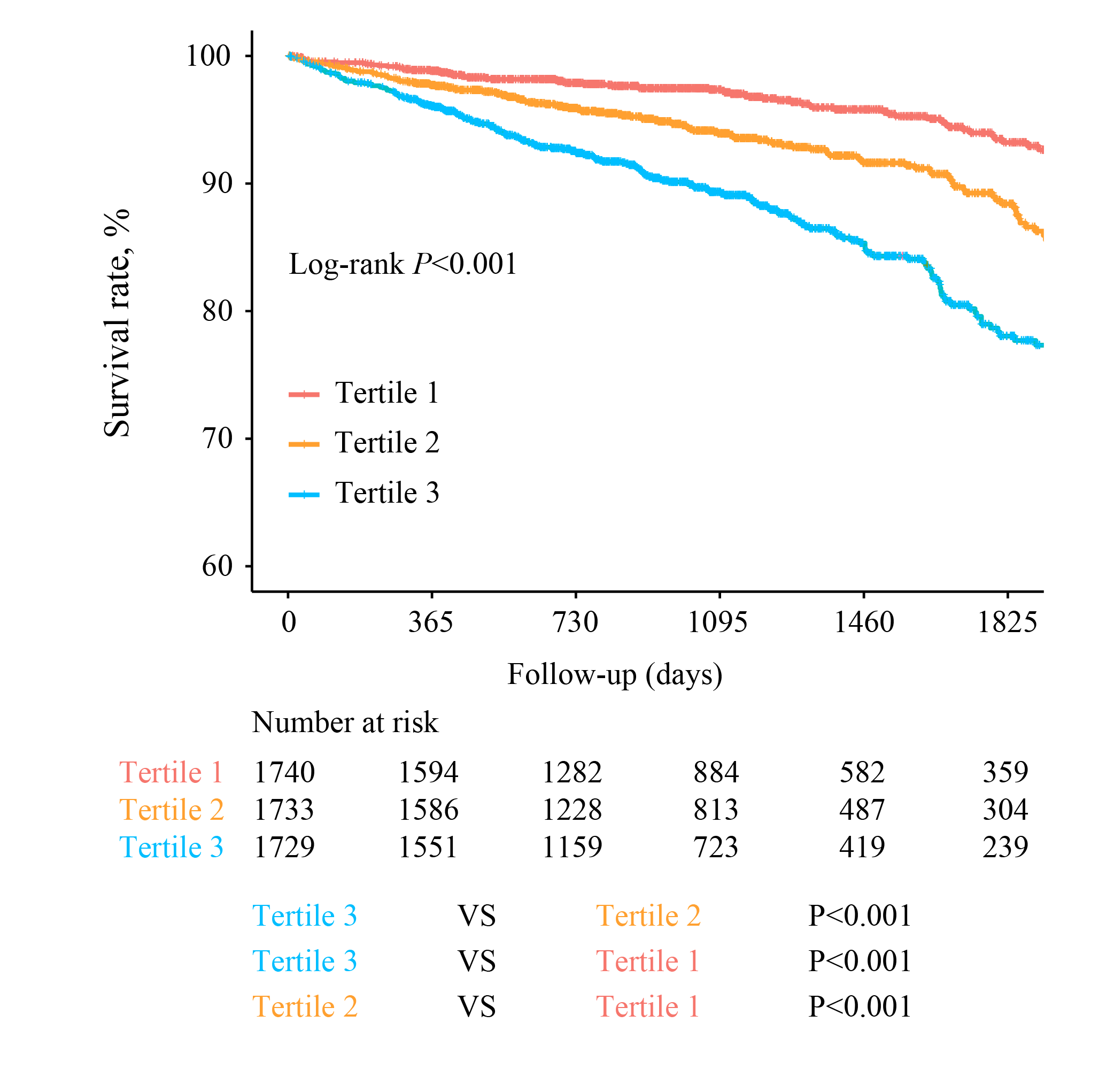

Supplement: Supplementary file 4 — Supporting information. [file CLC-47-e24219-s001.tif]
