# Supplementary material for: Lactate dehydrogenase‐to‐albumin ratio: A superior inflammatory marker for predicting contrast‐associated acute kidney injury after percutaneous coronary intervention
Source: Clin Cardiol. 2024 Jan 29;47(2):e24219. doi: 10.1002/clc.24219 (PMC10823551; doi:10.1002/clc.24219)
Supplement: Supplementary file 5 — Supporting information. [file CLC-47-e24219-s005.docx]

**Supplementary material**

**Legends**

**Supplementary Table 1.** Common inflammatory biomarkers and their calculation formulas

**Supplementary Table 2.** Correlation between LAR and other inflammatory markers

**Supplementary Table 3.** Baseline characteristics of patients with and without CA-AKI

**Supplementary Table 4.** Comparative analysis of the AUCs for predicting CA-AKI using LAR and other inflammatory markers

**Supplementary Table 5.** The odds ratio for the association of LAR with CA-AKI (defined by ESUR)

**Supplementary Figure 1.** Study flow chart.

**Supplementary Figure 2.** The incidence of CA-AKI among three groups.

**Supplementary Figure 3.** Subgroup analysis of the LAR and risk of CA-AKI.

**Supplementary Figure 4.** Kaplan-Meier curves for all-cause mortality according to LAR tertiles.

**Supplementary Table 1.** Common inflammatory biomarkers and their calculation formulas.

| Inflammatory biomarkers | Calculation formulas |
| --- | --- |
| SII | (neutrophil count × platelet count) / lymphocyte count |
| SIRI | (neutrophil count × monocyte count) / lymphocyte count |
| NLR | neutrophil count / lymphocyte count |
| dNLR | neutrophil count / (white blood cell count - neutrophil count) |
| NPAR | neutrophil percentage / albumin |
| PLR | platelet count / lymphocyte count |
| MLR | monocyte count / lymphocyte count |
| PNI | albumin + 5 × lymphocyte count |

**Abbreviations:** SII, systemic immune-inflammatory index; SIRI, systemic inflammation response index; NLR, neutrophil-to-lymphocyte ratio; dNLR, derived neutrophil-to-lymphocyte ratio; NPAR, neutrophil percentage-to-albumin ratio; PLR, platelet-to-lymphocyte ratio; MLR, monocyte-to-lymphocyte ratio; PNI, prognostic nutritional index.

**Supplementary table 2.** Correlation between LAR and other inflammatory markers.

| **Other inflammatory markers** | **LAR** | |
| --- | --- | --- |
|  | **Correlation coefficient** | ***P* value** |
| SII | 0.340 | <0.001 |
| SIRI | 0.474 | <0.001 |
| NLR | 0.392 | <0.001 |
| dNLR | 0.360 | <0.001 |
| NPAR | 0.332 | <0.001 |
| PLR | 0.141 | <0.001 |
| MLR | 0.381 | <0.001 |
| PNI | -0.273 | <0.001 |

**Abbreviations:** Abbreviations were shown in Supplement Table 1.

**Supplementary Table 3.** Baseline characteristics of patients with and without CA-AKI.

|  | **Without CA-AKI** | **With CA-AKI** | ***P* value** |
| --- | --- | --- | --- |
| Number of patients | n=5120 | n=315 |  |
| **Demographic information** | | | |
| Age, mean ± SD, years | 65.2 ± 10.3 | 68.1 ± 11.2 | <0.001 |
| Age >75 years, n (%) | 848 (16.7) | 88 (27.9) | <0.001 |
| Sex, female, n (%) | 1083 (21.2) | 90 (28.6) | 0.002 |
| BMI, median (IQR), kg/m^2^ | 24.2 (22.3-26.3) | 24.2 (22.3-26.1) | 0.243 |
| **Comorbidities, n (%)** | | | |
| Diabetes mellitus | 1807 (35.3) | 135 (42.9) | 0.008 |
| Hypertension | 3454 (67.5) | 239 (75.9) | 0.002 |
| Congestive heart failure | 198 (3.9) | 43 (13.7) | <0.001 |
| Chronic kidney disease | 428 (8.4) | 59 (18.7) | <0.001 |
| Anemia | 433 (8.5) | 71 (22.5) | <0.001 |
| AMI | 1473 (28.8) | 184 (58.4) | <0.001 |
| Hypotension | 324 (6.3) | 42 (13.3) | <0.001 |
| **Laboratory tests, median (IQR)** | | | |
| White blood cell, 10^9^/L | 6.94 (5.79-8.34) | 7.37 (6.00-9.34) | <0.001 |
| Hemoglobin, g/L | 139 (128-149) | 131 (119-144) | <0.001 |
| Platelet, 10^9^/L | 213 (181-254) | 211 (179-246) | 0.390 |
| Neutrophil, 10^9^/L | 4.26 (3.37-5.44) | 4.93 (3.61-6.73) | <0.001 |
| Neutrophil percentage, % | 64.1 (57.7-70.6) | 66.8 (61.5-73.1) | <0.001 |
| Monocyte, 10^9^/L | 0.46 (0.36-0.59) | 0.52 (0.38-0.64) | <0.001 |
| Lymphocyte, 10^9^/L | 1.84 (1.47-2.31) | 1.68 (1.35-2.13) | <0.001 |
| Creatinine, mg/dL | 0.88 (0.76-1.02) | 0.86 (0.71-1.06) | 0.386 |
| eGFR, mL/min/1.73m^2^ | 92 (78-100) | 89 (67-100) | 0.024 |
| LDH, U/L | 173 (151-210) | 203 (170-340) | <0.001 |
| Albumin, g/L | 42 (39-44) | 39 (36-42) | <0.001 |
| Cholesterol, mmol/L | 4.05 (3.41-4.89) | 4.15 (3.50-5.06) | 0.124 |
| LDL-C, mmol/L | 2.56 (2.00-3.28) | 2.68 (2.03-3.34) | 0.122 |
| Glucose, mmol/L | 5.46 (4.93-6.53) | 5.77 (5.06-7.22) | <0.001 |
| HbA1c, % | 6.2 (5.8-7.0) | 6.3 (5.9-7.6) | 0.004 |
| D-dimer, mg/L FEU | 0.32 (0.19-0.63) | 0.54 (0.26-0.96) | <0.001 |
| NT-proBNP, pg/mL | 182 (66-666) | 1111 (294-2940) | <0.001 |
| LVEF <40, n (%) | 120 (2.5) | 20 (6.6) | <0.001 |
| LVEF, % | 59 (57-62) | 57 (48-60) | <0.001 |
| LAR | 4.11 (3.53-5.18) | 5.45 (4.19-9.21) | <0.001 |
| SII | 485 (345-715) | 620 (419-942) | <0.001 |
| SIRI | 1.04 (0.68-1.64) | 1.45 (0.88-2.34) | <0.001 |
| NLR | 2.27 (1.68-3.18) | 2.84 (1.98-4.05) | <0.001 |
| dNLR | 1.66 (1.28-2.23) | 2.01 (1.54-2.75) | <0.001 |
| NPAR | 15.34 (13.50-17.39) | 17.17 (15.13-19.47) | <0.001 |
| PLR | 115 (91-18) | 122 (97-165) | 0.001 |
| MLR | 0.25 (0.18-0.34) | 0.30 (0.21-0.41) | <0.001 |
| PNI | 51 (47-55) | 48 (44-52) | <0.001 |
| **Medication use during hospitalization, n (%)** | | | |
| Antiplatelet agents | 5113 (99.9) | 313 (99.4) | 0.092 |
| Statin | 5078 (99.2) | 312 (99.1) | 0.744 |
| ACEI/ARB | 4222 (82.5) | 273 (86.7) | 0.066 |
| β-blocker | 4223 (82.5) | 272 (86.4) | 0.092 |
| Diuretics | 1148 (22.4) | 189 (60.0) | <0.001 |
| **Angiographic parameters, mean ± SD** | | | |
| Multivessel disease, n (%) | 4101 (83.2) | 259 (85.8) | 0.274 |
| Stent length, mm | 44.73 ± 26.69 | 46.79 ± 27.83 | 0.211 |
| Number of stents, n | 1.65 ± 0.86 | 1.69 ± 0.93 | 0.455 |

**Abbreviations:** BMI, body mass index; AMI, acute myocardial infarction; eGFR, estimated glomerular filtration rate; LDH, lactate dehydrogenase; LDL-C, low density lipoprotein-cholesterol; HbA1c (%), glycosylated hemoglobin; NT-proBNP, N-terminal pro-brain natriuretic peptide; LVEF, left ventricular ejection fraction; ACEI/ARB, angiotensin-converting enzyme inhibitor/angiotensin receptor blocker. other abbreviations were shown in Supplement Table 1.

**Supplementary Table 4.** Comparative analysis of the AUCs for predicting CA-AKI using LAR and other inflammatory markers.

| **Eight**  **inflammatory markers** | **AUC (95% CI)** | **ΔAUC** | **DeLong test**  ***P*-value** |
| --- | --- | --- | --- |
| LAR | 0.693 (0.662-0.723) | Reference |  |
| SII | 0.607 (0.574-0.640) | 0.086 | <0.001 |
| SIRI | 0.621 (0.589-0.654) | 0.072 | <0.001 |
| NLR | 0.625 (0.593-0.656) | 0.068 | <0.001 |
| dNLR | 0.624 (0.592-0.656) | 0.069 | <0.001 |
| NPAR | 0.659 (0.628-0.691) | 0.034 | 0.031 |
| PLR | 0.558 (0.525-0.591) | 0.135 | <0.001 |
| MLR | 0.601 (0.567-0.634) | 0.092 | <0.001 |
| PNI | 0.652 (0.620-0.684) | 0.041 | 0.015 |

**Abbreviations:** AUC, area under the curve; Other abbreviations were shown in Supplement Table 1.

**Supplementary Table 5.** The odds ratio for the association of LAR with CA-AKI (defined by ESUR).

|  | Each 1-SD increase | LAR Tertiles | | | *P* for trend |
| --- | --- | --- | --- | --- | --- |
|  |  | Tertile 1 | Tertile 2 | Tertile 3 |  |
| Unadjusted model | 1.31 (1.22-1.41) *** | 1 (Ref.) | 1.31 (1.10-1.57) ** | 2.11 (1.78-2.50) *** | <0.001 |
| Model 1 | 1.33 (1.24-1.44) *** | 1 (Ref.) | 1.24 (1.03-1.49) * | 2.02 (1.70-2.40) *** | <0.001 |
| Model 2 | 1.32 (1.23-1.43) *** | 1 (Ref.) | 1.26 (1.05-1.51) * | 2.05 (1.72-2.45) *** | <0.001 |
| Model 3 | 1.19 (1.10-1.29) *** | 1 (Ref.) | 1.22 (1.01-1.48) * | 1.50 (1.22-1.85) *** | <0.001 |

**Notes:** * *P*<0.05, ** *P*<0.01, *** *P*<0.001.

Model 1: adjusted age >75 years and sex.

Model 2: adjusted for Model 1 + diabetes mellitus, hypertension, chronic kidney disease, congestive heart failure, and hypotension.

Model 3: adjusted for Model 2 + acute myocardial infarction, anemia, and contrast media >150 mL.

**Abbreviations:** LAR, dehydrogenase-to-albumin ratio; CA-AKI, contrast-associated acute kidney injury.

**Supplementary Figure 1.** Study flow chart.

**
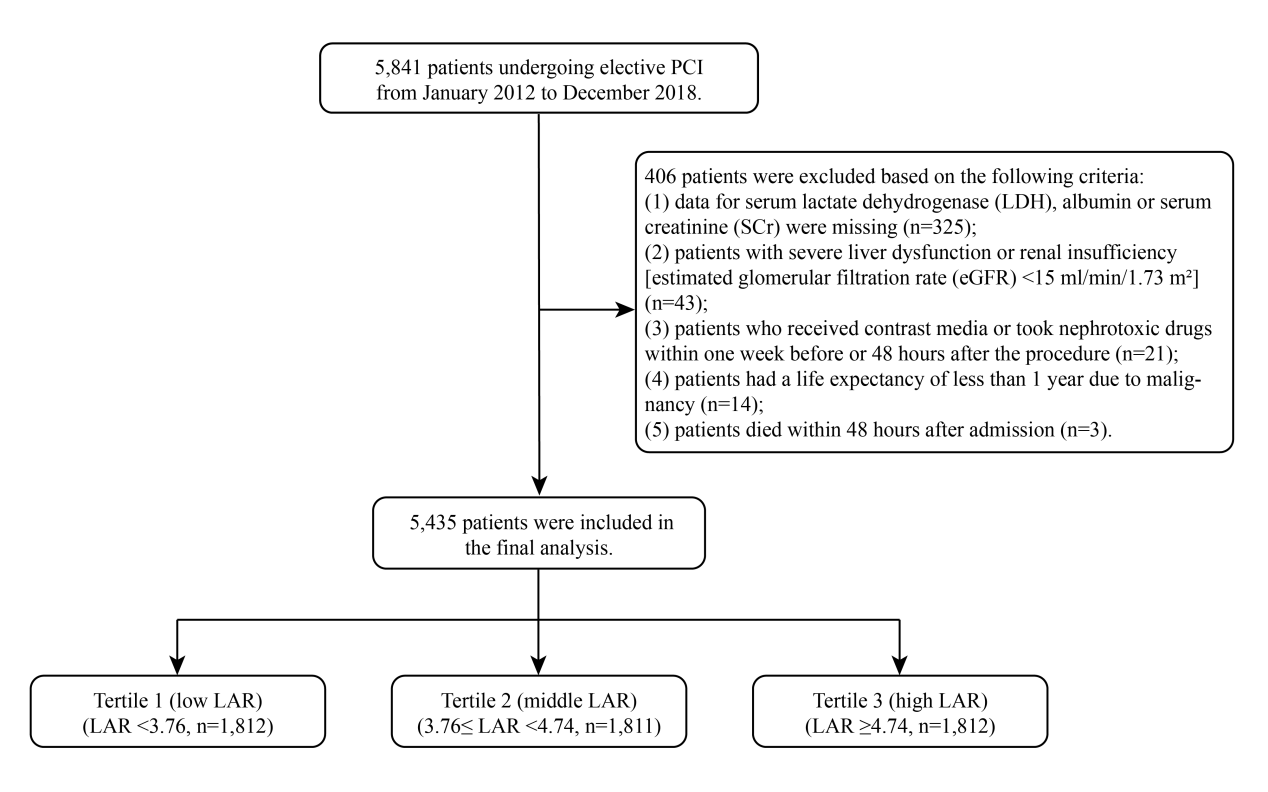
Abbreviations:** LAR, dehydrogenase-to-albumin ratio; PCI, percutaneous coronary intervention.

**Supplementary Figure 2.** The incidence of CA-AKI among three groups.


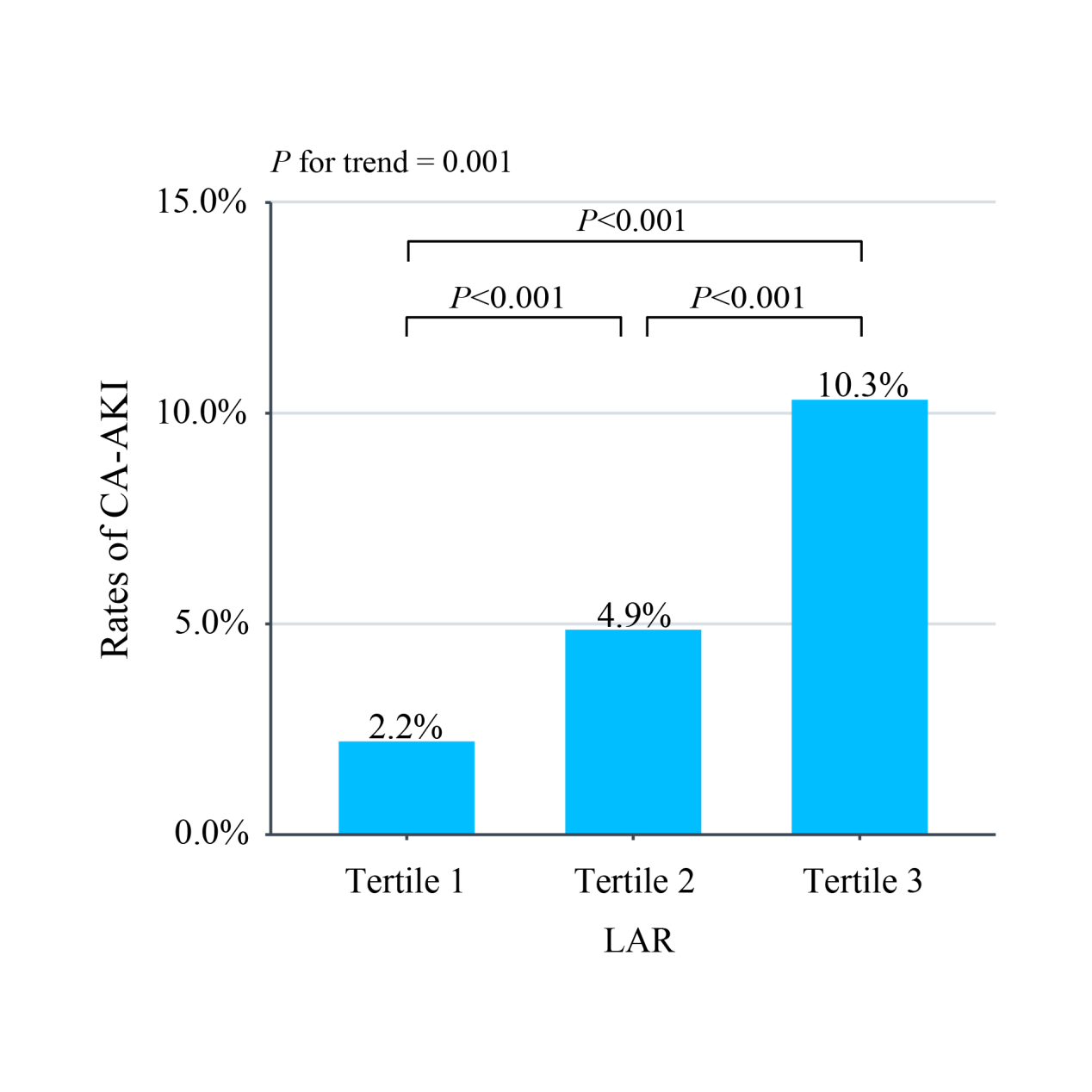


**Abbreviations:** LAR, dehydrogenase-to-albumin ratio; CA-AKI, contrast-associated acute kidney injury.

**Supplementary Figure 3.** Subgroup analysis of the LAR and risk of CA-AKI.


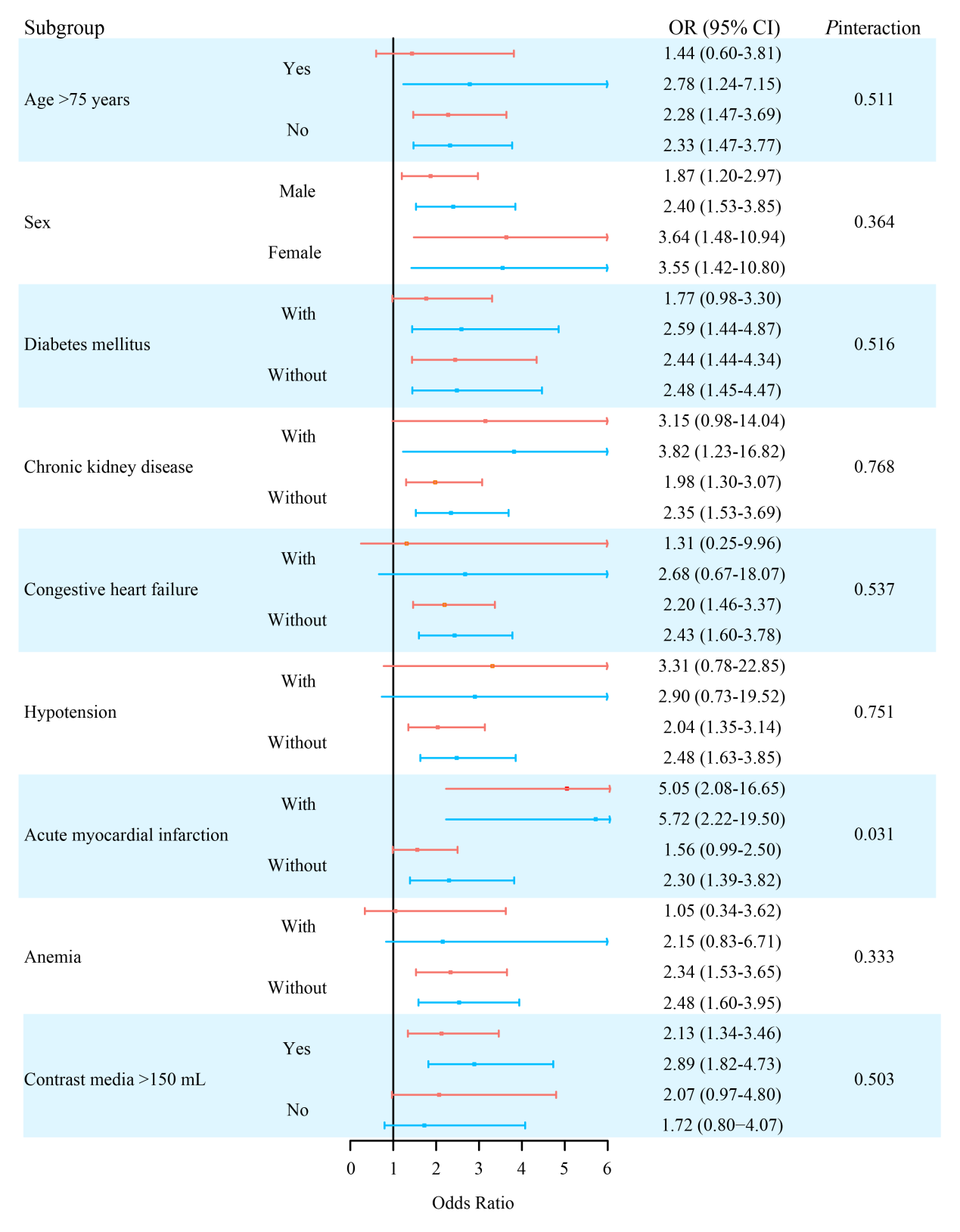


**Notes:** The red line represents Tertile 2, while the blue line represents Tertile 3.

**Abbreviations:** LAR, dehydrogenase-to-albumin ratio; CA-AKI, contrast-associated acute kidney injury.

**Supplementary Figure 4.** Kaplan-Meier curves for all-cause mortality according to LAR tertiles.


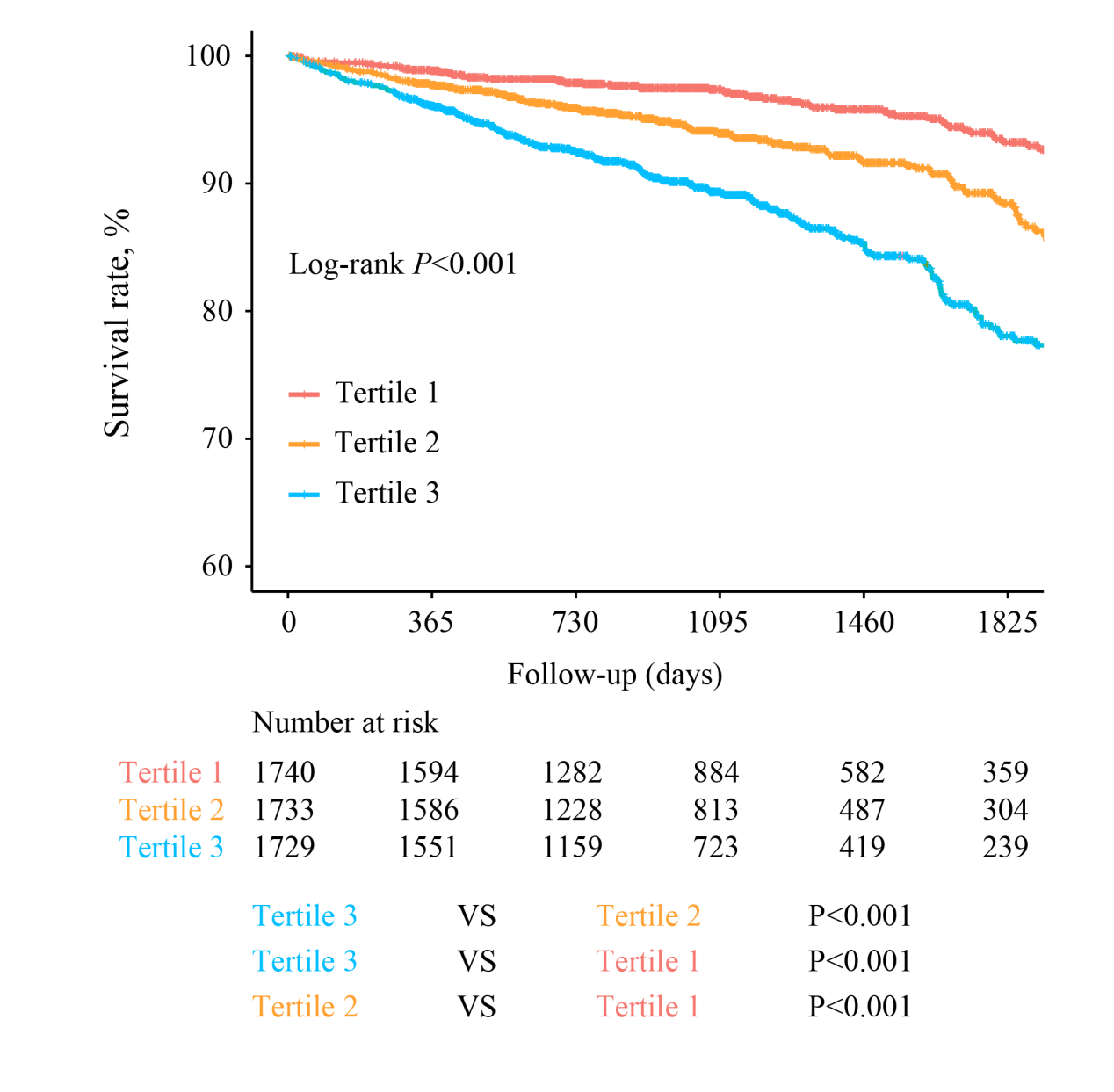


**Abbreviations:** LAR, dehydrogenase-to-albumin ratio.
